# Can mechanical imaging increase the specificity of mammography screening?

**DOI:** 10.1007/s00330-016-4723-6

**Published:** 2017-01-20

**Authors:** Magnus Dustler, Daniel Förnvik, Pontus Timberg, Ingvar Andersson, Hannie Petersson, Håkan Brorson, Anders Tingberg, Sophia Zackrisson

**Affiliations:** 1 Medical Radiation Physics, Department of Translational Medicine, Faculty of Medicine, Lund University, Skåne University Hospital, SE-205 02 Malmö, Sweden; 2 Mammography Clinic, Unilabs AB, Jan Waldenströms Gata 22, SE-205 02 Malmö, Sweden; 3 Plastic and Reconstructive Surgery, Department of Clinical Sciences Malmö, Faculty of Medicine, Lund University, Skåne University Hospital, SE-205 02 Malmö, Sweden; 4 Diagnostic Radiology, Department of Translational Medicine, Faculty of Medicine, Lund University, Skåne University Hospital, SE-205 02 Malmö, Sweden

**Keywords:** Mechanical imaging, Mammography, Breast screening, Recall rates, Biopsy rate

## Abstract

**Objectives:**

This study aimed to investigate the effects of adding adjunct mechanical imaging to mammography breast screening. We hypothesized that mechanical imaging could detect increased local pressure caused by both malignant and benign breast lesions and that a pressure threshold for malignancy could be established. The impact of this on breast screening was investigated with regard to reductions in recall and biopsy rates.

**Methods:**

155 women recalled from breast screening were included in the study, which was approved by the regional ethical review board (dnr 2013/620). Mechanical imaging readings were acquired of the symptomatic breast. The relative mean pressure on the suspicious area (RMPA) was defined and a threshold for malignancy was established.

**Results:**

Biopsy-proven invasive cancers had a median RMPA of 3.0 (interquartile range (IQR) = 3.7), significantly different from biopsy-proven benign at 1.3 (IQR = 1.0) and non-biopsied cases at 1.0 (IQR = 1.3) (P < 0.001). The lowest RMPA for invasive cancer was 1.4, with 23 biopsy-proven benign and 33 non-biopsied cases being below this limit. Had these women not been recalled, recall rates would have been reduced by 36% and biopsy rates by 32%.

**Conclusions:**

If implemented in a screening situation, this may substantially lower the number of false positives.

***Key Points*:**

• *Mechanical imaging is used as an adjunct to mammography in breast screening.*

• *A threshold pressure can be established for malignant breast cancer.*

• *Recalls and biopsies can be substantially reduced.*

## Introduction

### Mammography screening recalls

Mammography is the premier means of breast cancer screening worldwide and is, despite some criticism, considered an effective way of reducing breast cancer mortality [1–3]. Screening incurs significant expenses for the healthcare system [4–6]. According to the European Guidelines for Quality Assurance in Mammography Screening [7] recall rates should be 3–5% (7% in the prevalence round). A recent publication reported recall rates in three countries – the USA, Norway and Spain – to be 9.1%, 3.2% and 4.2%, respectively [8]. The rate of screening detected cancers was 0.4–0.55% per screening round, meaning that about 90% of recalls are false positives. Another study estimated that of women screened biennially starting at the age of 50 years, 20% would have a false positive result at least once before the age of 68 years [9]. False positives put a substantial economic demand on the healthcare system, and subject women to considerable anxiety and other negative psychosocial consequences [10–13].

False positives can be divided into two groups: benign findings and normal tissue. The first group consists of several types of benign lesions – cysts, fibroadenoma, papillomas etc., while the second is normal tissue which appears suspicious, e.g. over-projection of fibroglandular strands at different depths.

### Other breast imaging modalities

Breast tomosynthesis has the potential to complement and/or replace digital mammography in standard screening practice. European prospective studies and US retrospective studies have investigated its impact on breast cancer screening [14–23]. Three prospective studies – STORM [14], OTST [18] and MBTST [23] – have shown superior cancer detection. Concerning recall rates, the prospective studies show an increase, while the retrospective studies generally show a decrease [15–17, 19–22]. Spectral mammography is another alternative, with a recent study demonstrating its ability to distinguish between cystic and solid lesions [24].

In addition to mammography, ultrasonography is often used in the work-up of recalled women. It is effective at differentiating malignant and benign findings [25–27]. General screening with ultrasound is not implemented due to a lack of cost-effectiveness, related to examination time and the need for a trained operator to carry out the procedure, and the high false-positive rate [28–30]. The J-START trial investigated ultrasonography as an adjunct to mammography in screening on 72,988 women, and found it to be effective for detecting more cancers at an earlier stage, but did not assess its economic efficacy [31–33]. Various automatic and semi-automatic ultrasound systems exist, but their performance in screening has not been established [34].

### Mechanical imaging

There are marked differences in the mechanical properties of various types of breast tissue [35]. The modulus of elasticity, or Young’s modulus – for an elastic material subject to a certain degree of deformation – relates the stress (pressure exerted on the material) to the strain (relative deformation of the material), i.e. the greater the elastic modulus, the stiffer the material. The tissues in the human breast are non-linearly elastic, which means that the value of the elastic modulus increases with the level of strain.

Mechanical imaging (MI) is defined as the practice of deforming tissue and measuring the resulting distribution of pressure – or stress field – using pressure sensors [36, 37]. The measured pressures on different locations provide information about the underlying elastic modulus of the deformed tissue, as the pressure will be proportionately higher with increasing elastic modulus. As there is a considerable difference in elastic modulus between various types of benign and malignant breast tissue there is thus the possibility of using this technique to differentiate such structures. Notably, according to Krouskop et al., malignant tumours have a substantially greater elastic modulus than other tissues, especially so at higher degrees of deformation (strain) [35]. Egorov et al. described a sensitivity of 91% and specificity of 87% for a form of MI of the breast [38, 39]. This method uses a handheld probe – similar in appearance to an ultrasound probe – which is manually moved over the parts of the breast being examined.

Our group has in earlier studies used a Tekscan Iscan force sensing resistor system (Tekscan Inc., South Boston, MA, USA) consisting of pressure sensors attached to the compression plate of a mammography device to obtain pressure readings of the compressed breast [40–43]. In one study we investigated the pressure over cancerous lesions, finding a significant difference in pressure over the lesions compared to the background pressure [43]. The use of pressure sensors during imaging at mammography screening, giving real-time pressure reading in conjunction with the screening images, has not previously been investigated.

### Aims

This study aimed to use MI to investigate if it is possible to differentiate malignant lesions from benign and normal findings in the female breast, and what effect using such a procedure as an additional modality to screening mammography would have on recall rates.

## Methods and materials

### Study design

The experimental setup used MI on women recalled from screening. A ‘minimum threshold for malignancy’ was established by correlation of local pressure with pathology. From this, the impact of adding adjunct MI to screening was estimated in terms of reduced recall rates, assuming that the radiologist would recommend recall based on the screening mammogram and mark the location of the suspicious finding, with an actual recall assumed to be made only if the local pressure on that feature exceeded the threshold for malignancy.

### Data acquisition

**Fig. 1:**
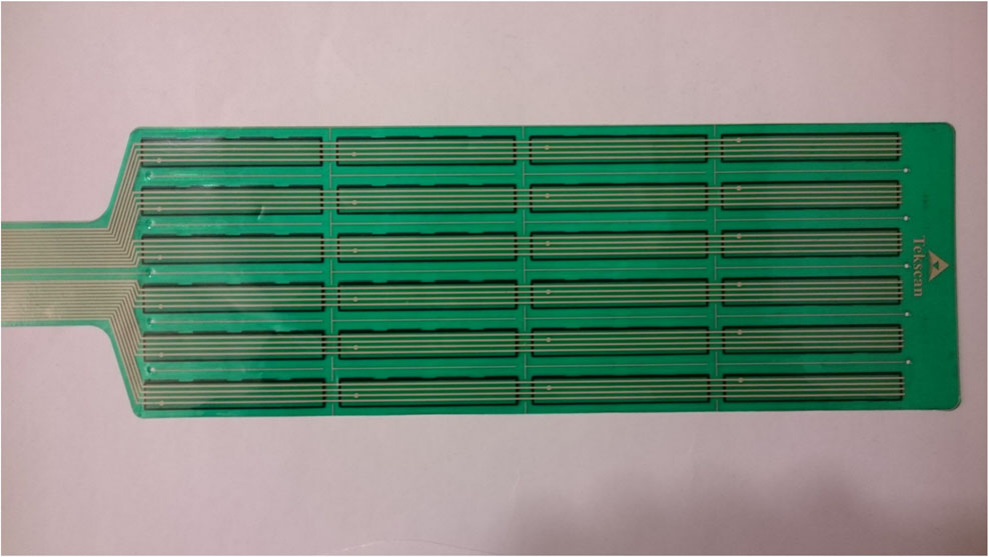
A Tekscan Iscan 9801 pressure sensor employed in the study. Each strip consists of four sensor elements. The electrical impedance of the sensor elements decreases when subject to pressure

An improvised MI device was created by affixing two Iscan model 9801 sensors to the inferior side of the compression paddle of a MAMMOMAT Inspiration mammography device (Siemens Healthcare GmbH, Erlangen, Germany). These sensors have been used in our previous studies, and their performance in similar situations has been investigated [44, 45]. Each sensor consists of 96 individual sensor elements, arrayed in six rows of 16. Each sensor element is a square with a side of 12.7 mm and a total thickness of roughly 0.16 mm (Fig. 1).

**Fig. 2:**
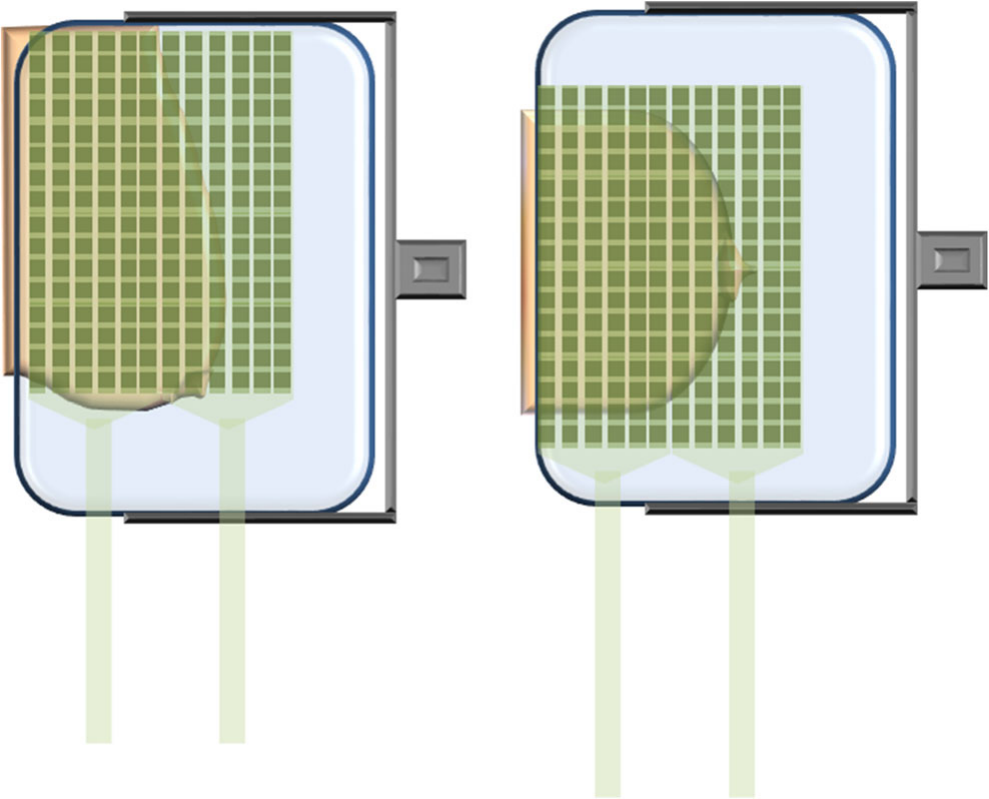
Illustration of sensor positioning on the compression paddle. The two sensor matrices were positioned adjacent to each other to cover as much of the compression paddle as possible. For the craniocaudal (CC) view, the sensors were centred on the juxta-thoracic edge of the compression paddle (right). For the mediolateral oblique (MLO) view, sensors were instead placed in contact with the appropriate axillary edge of the paddle, in order to cover axillary tissue (left)

The sensors were positioned in contact with the juxta-thoracic edge of the compression paddle, so they could measure pressure as closely as possible to the chest wall. To maximize breast coverage, for the cranio-caudal (CC) projection the sensors were centred on the paddle, while on the mediolateral oblique (MLO) projection they were positioned in contact with the appropriate axillary edge (Fig. 2). The projection used was individually determined based on which one was considered to be most likely to have pressure distributed to the suspicious area, with CC preferred in unclear cases.

MI was carried out subsequent to mammography of the recalled woman. The breast was positioned as normal for the corresponding mammography projection and compressed to the standard level of compression. Pressure readings from both sensors were acquired in sequence. A low dose mammogram (5 mAs) of the breast with attached sensors was also acquired, in order to be able to match pressure data with radiological findings.

### Data analysis

The centre of each suspicious feature was determined by comparing the clinical mammogram of each case with the corresponding minimal-dose mammogram with included sensors. The mean pressure of the 3 × 3 sensor elements centred on each feature was measured and then normalized by the mean pressure over the breast, defined as the mean pressure of all sensor elements completely covered by the breast as determined by inspecting the minimal-dose mammogram. This value was referred to as RMPA (relative mean pressure over lesion area). For women having multiple suspicious areas, the highest individual RMPA was used. The column of the sensor matrix closest to the chest wall, and for MLO-cases the row closest to the armpit, were excluded because of the pressure values in these areas frequently being saturated.

Wilcoxon’s rank sum test was used to compare the median values of groups, with significant differences defined at the 95% confidence level. Descriptive statistics given in the *Results* section are thus the median and interquartile range (IQR) of various groups of data.

### Study population

Enrolled women were from those participating in the Swedish national breast screening program at Skåne University Hospital in Malmö, between 27 February 2014 and 11 March 2015. The study was approved by the regional ethical review board (dnr 2013/620). A notification that they might be asked to take part in a study involving pressure measurements was included in the recall notification.

A number of time slots were set-aside each week for study patients depending on the availability of the researchers and the needs of the clinic. All recalled women scheduled for those time slots received additional written and oral information about the study upon arrival and were asked to provide informed consent to participate in the study. The scheduling of examinations deliberately excluded stereotactic biopsies, in order not to interrupt the clinical workflow. In addition, non-Swedish speaking women as well as women with breast implants were excluded.

The study involved 155 women. Screening notes and primary radiological reports were used to identify the suspicious areas that warranted recall of the woman. In unclear cases, all women were considered to have at least one suspicious feature: (1) if no specific area was mentioned as the reason for recall but this was still obvious from later clinical mammograms and/or other sources, or (2) if no suspicious area could be identified. If multiple suspicious areas were identified, the one with the highest RMPA was used.

### Validation of measurements

In order to identify potentially inconclusive readings, all MI-readings were validated based on five criteria before determination of RMPA:

1. Technical problems with sensors, measurement electronics and/or the measurement software
2. Feature not in the field-of-view in the projection in which pressure measurements were acquired
3. Feature located in an area with very high or saturated pressure values, i.e. on the pectoral muscle or on the column of pressure sensors closest to the chest wall
4. Feature not present on recall
5. Very low or no pressure on the location of the feature, i.e. the feature being either in the periphery of the breast – where there is no pressure as there is no contact with the compression paddle – or in a part of the breast lacking compression due to unfavourable pressure distribution.

Any woman meeting one of these criteria was automatically included in the recalled group, just as if the RMPA had been higher than the recall threshold as malignancy could not be ruled out based on MI.

## Results

Biopsies were carried out on 71 women, of which 50 were benign, representing either benign lesions or normal tissue. These are labelled *biopsy-proven benign*. The remaining 84 cases did not warrant a biopsy and were thus very likely (but not certainly) benign. These are labelled as *other benign*. The biopsy-proven cancers are labelled as such (Table 1).

For a number of women no useful MI-readings could be acquired, according to the five criteria described above (Table 2). The conclusive cases consisted of: 14 *biopsy-proven malignant lesions* (11 IDC, ILC or tubular, two DCIS (DCIS only, no other signs of malignancy, i.e. discernible mass) and one non-Hodgkin’s lymphoma), *43 biopsy-proven benign*, 53 *other benign*.

**Table 1:** Overview of the inconclusive cases, and the criteria identifying them

	Biopsy-proven malignant	Biopsy-proven benign	Other benign	All
Total	21	50	84	155
Total inconclusive	7	7	31	45
Technical problems	2	4	5	11
Outside field-of-view	2	2	7	11
On pectoral muscle/chest wall	0	0	3	3
Not present at recall	0	0	9	9
Low pressure on suspicious area	3	1	7	11

**Table 2:** Description of biopsy results for suspicious biopsied findings

Biopsy results	Number
Malignant (biopsy-proven malignant)	21
Invasive ductal carcinoma	11
Invasive lobular carcinoma	5
Tubular carcinoma	1
Ductal carcinoma in situ	2
Non-Hodgkin’s lymphoma	2
Benign (biopsy-proven benign)	50
Not biopsied (other benign)	84

For *biopsy-proven benign* features RMPA values were found to be 1.3 (IQR = 1.0), which was not significantly different (P = 0.63) from the RMPA values for *other benign* features, 1.0 (IQR = 1.1). After excluding the remaining cases of DCIS (2) and non-Hodgkin’s (1) lymphoma the value became 3.0 (IQR = 3.7) for *biopsy-proven cancers*. The two DCIS cases had RMPA values of 0.6 and 0.9, respectively, while the single non-Hodgkin’s lymphoma case had a value of 0.7. The RMPA of the cancer group was significantly different from both the *biopsy-proven benign* cases (P < .0001) and *other benign* cases (P < .0001).

**Fig. 3:**
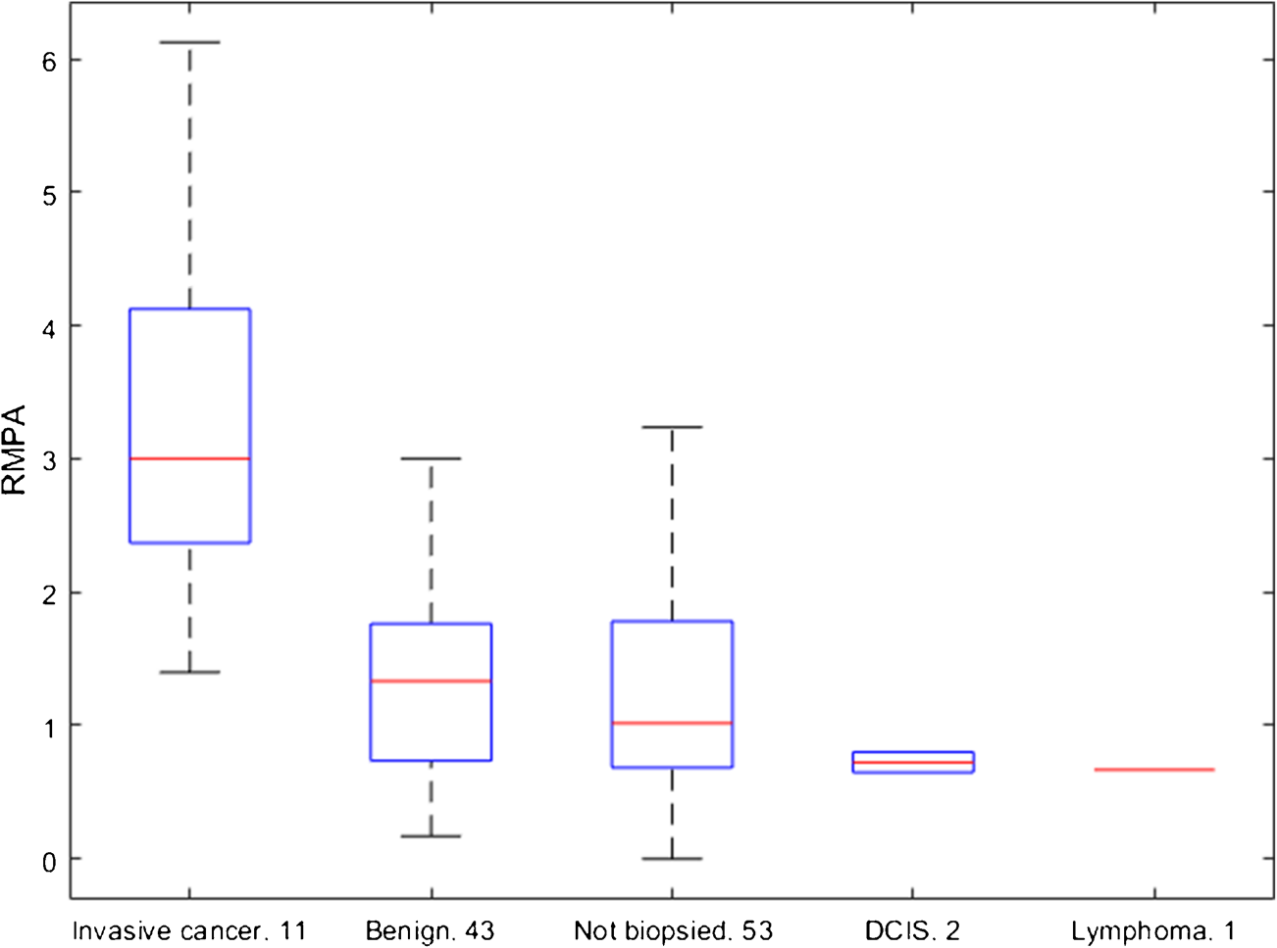
Differences in relative mean pressure (RMPA) between the included groups. Note that ductal carcinoma in situ (DCIS; in isolation, i.e. not associated with any mass) lies close to the mean breast pressure. There is no significant difference between the biopsied and non-biopsied groups of benign lesions, though both benign groups lie significantly under the malignant group

**Fig. 4:**
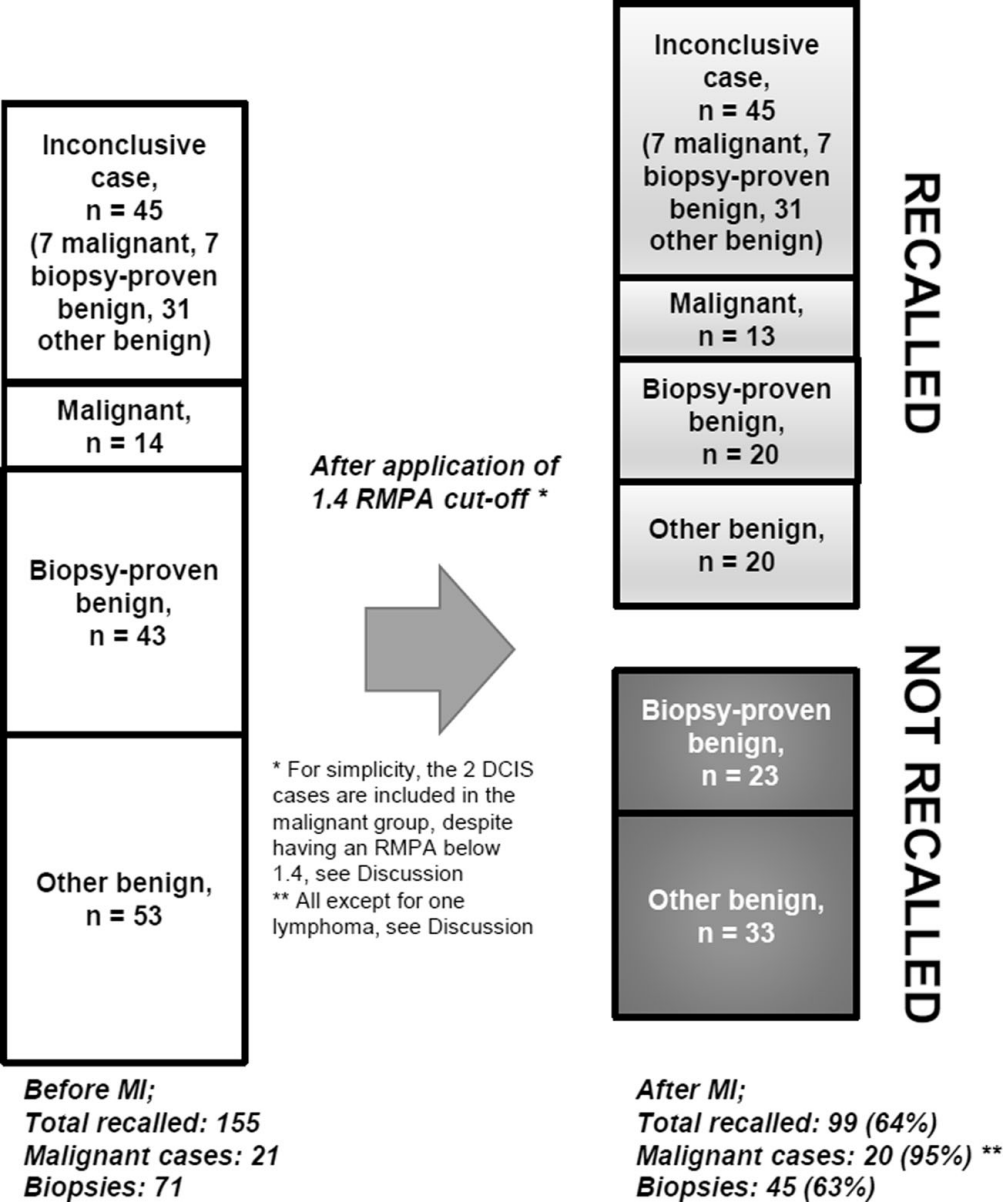
Overview of the effect on recalls after taking mechanical imaging into account; 38% of the total number of cases do not require recall, including 32% of those biopsied. Note that the inconclusive cases are included in the total number of recalls, as MI cannot be used to draw any conclusions in those cases

**Fig. 5:**
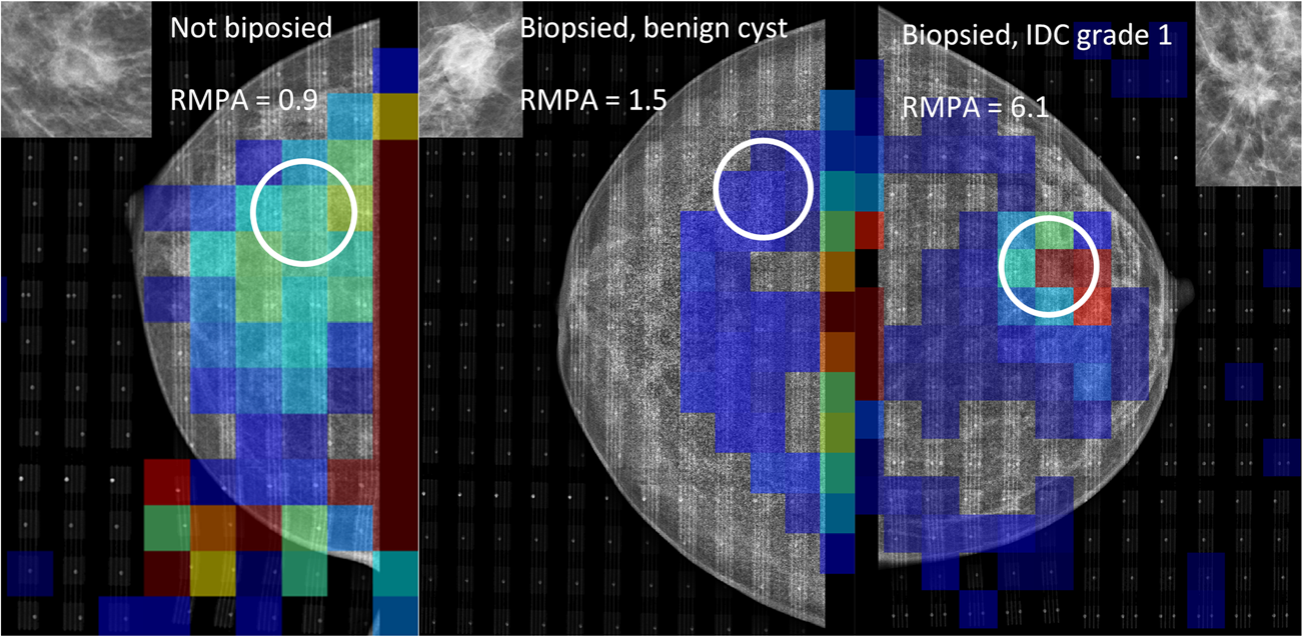
Examples of mechanical imaging data from the study, matched with corresponding mammogram. All three groups are represented, from left to right: *A*, not biopsied, presumed benign; *B*, biopsied, benign; and *C*, biopsied, malignant. The relative mean pressure over lesion area (RMPA) is defined as the mean of the pressure values in the 3 × 3 sensor elements centred on the suspicious feature, normalized by mean pressure over the breast. Note the presence of a high pressure area outside of the breast on the leftmost image; it is caused by wrinkling of the sensor. This is one example of a technical problem, which in this case did not affect the measurement

Including all malignant cases, the minimum RMPA for malignancy was 0.6, while excluding DCIS and non-Hodgkin’s lymphoma put the minimum threshold at 1.4 (Fig. 3). Using the lower level, this would put 18 benign cases below the recall limit, nine of which were biopsied. With the higher limit, 56 benign cases were below the limit, 23 of which were biopsied. With the original population of 155 women, of which 71 were biopsied, this would be equivalent to a reduction of recall rates by 12% and 36%, respectively, with the biopsy rates likewise decreasing by 13% and 32%, respectively (Fig. 4). All women with inconclusive MI results would be recalled as normal. Examples of MI cases (malignant and benign) are shown in Fig. 5.

## Discussion

The results suggest a definable lower RMPA threshold for malignant breast cancer, below which all suspicious findings were benign. This is consistent with our hypothesis and earlier results in the field. The cancer material is rather small, which necessitates a larger study to establish the method’s applicability. That said, results show a substantial difference between malignant and benign cases. Though it is prudent to use a somewhat lower threshold, the benefits – both for the healthcare system and for patient comfort and compliance – would still be considerable. A reduction of recalls by almost 40% is similar to the most optimistic clinical appraisals of the effect of breast tomosynthesis screening [15, 16, 21, 22]. MI could be used alongside breast tomosynthesis as well as mammography, though the effects need to be investigated. Erhard et al. investigated using spectral mammography to avoid recalls by distinguishing fluid-filled cysts from malignant lesions [24]. If all recalls of fluid-filled cysts could be avoided, the total decrease of recalls would be 20%, with less than one missed cancer per 625 correctly identified cysts. A similar analysis of the MI system would be valuable in the future.

The calculations of recall and biopsy rate reductions assumed that all cases for which MI-readings could not be used in the final analysis would be recalled as indicated by screening mammography. The estimated reductions are thus conservative. In a screening implementation, the number of inconclusive measurements would probably be substantially lower. Technical problems were unavoidable with the basic prototype setup used in the study, with some cases excluded due to poor pressure over the suspicious feature likely resulting from this. The 11 cases that were inconclusive because of the suspicious feature not being present on MI could also presumably have been avoided. Other inconclusive measurements are likely unavoidable to some extent, as regardless of positioning, tumours in certain locations might never have substantial pressure applied to them due to the structure of the breast and the design of the compression paddle. This could be mitigated by performing MI in both MLO and CC with a greater chance of the suspicious feature being adequately compressed in at least one projection.

**Fig. 6:**
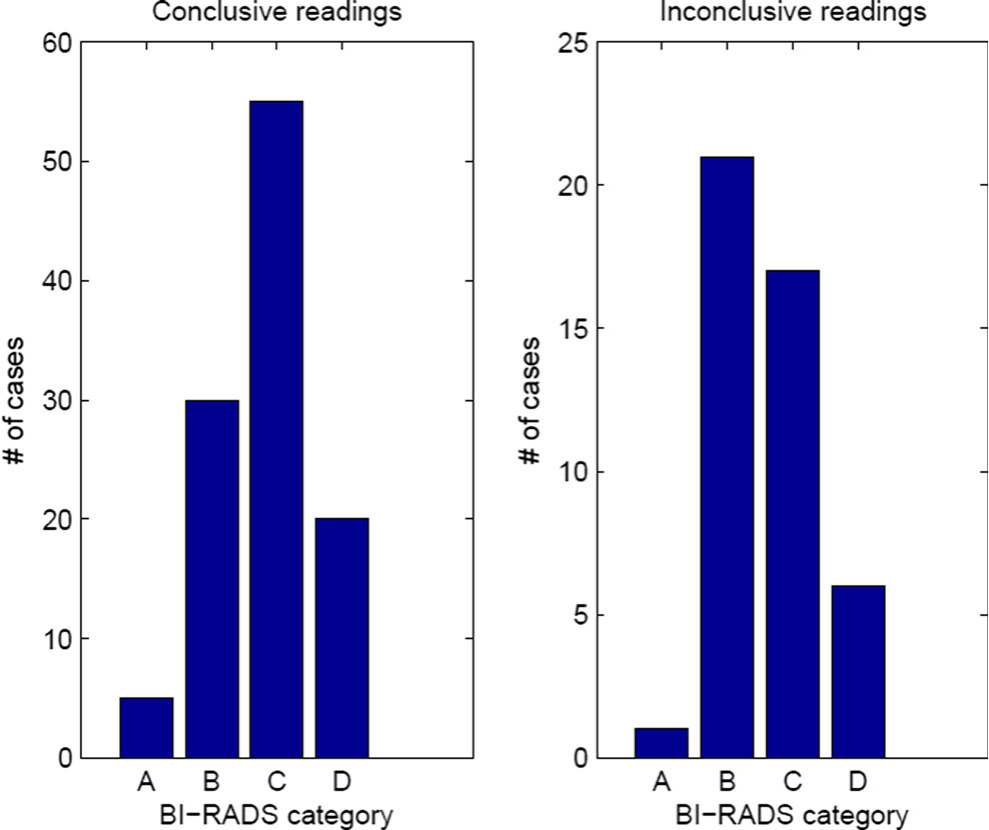
Bar plots of the BI-RADS density classifications of women included in the study. Women who had inconclusive readings are shown on the right. There was a trend towards the inconclusive group having a greater proportion of category B and lesser proportion of category C breasts, though this was not significant (P = .09)

The amount of inconclusive readings (29% overall) was similar for malignant cases (33%) and benign cases (28%), which implies that there was no bias. There was a trend towards more BI-RADS category B and less category C women among inconclusive cases, though this was not statistically significant (Fig. 6). Biopsied cases were less likely to be inconclusive than non-biopsied ones (20% vs. 37%). One criterion was that the suspicious finding did not present on recall, which partly explains this, as these were of course all in the non-biopsied group. Having MI data available for those cases in particular would be very valuable, as they are very likely to amount to over-projection of tissue.

Stereotactic biopsies are primarily performed on women with suspicious microcalcifications. This might make the number of DCIS cases disproportionately low.

One obstacle to the system’s clinical implementation is the sensors, which are clearly visible on the mammogram and impair image quality. To be effective, radio-translucent sensors are likely needed, or alternatively some form of subtraction imaging.

In the current study we only investigated effects on screening specificity, i.e. trying to reduce recalls. The mechanical imaging data was evaluated after identifying suspicious findings on mammography, to filter out false positives. It would also be of great value to investigate effects on sensitivity, i.e. using mechanical imaging to find lesions obscured on mammography. Variations in the normal pressure distribution would likely result in an excess of false positives. Possible remedies would be to correlate with the pressure distribution in the contralateral breast, and also with the distribution of dense tissue and BI-RADS density category. We intend to evaluate this in a further study on the collected material. Also a much larger prospective study is needed to fully investigate the method.

Boyd et al. investigated the relationship between breast tissue stiffness and risk of cancer, with results suggesting it to be an independent risk factor [46]. It might be that the elevated RMPA score of a malignant lesion is indicative of cancer not because the cancer increases local stiffness, but because areas with high stiffness have a higher chance of developing cancer. We speculate that cancer is more likely to develop in high stiffness areas (associated with increased local density) and alters stiffness both through its presence and through reactive fibrosis. Size, type and positioning also likely play a role, with RMPA being for example dependent on the size of the tumour in relation to the thickness of the breast. It is also likely that radiologically stellate and circumscribed lesions have different properties. In the study, all invasive breast cancers were stellate.

The material is too small to find MI differences between the various subtypes of invasive cancer. Ductal carcinoma in-situ are however distinct, with both cases in the study showing a non-elevated RMPA. This is not unexpected, as they expressed only as microcalcifications with no associated solid lesion. The MI system should thus be considered a screening detection aid for suspicious masses. When faced with suspicious looking clusters of microcalcifications radiologists must be prepared to recall despite MI readings. The two non-Hodgkin’s lymphoma in the material are a statistical anomaly. One lesion was located in the axillary area where no MI readings could be acquired, while the one located in the breast had a low RMPA, perhaps implying properties different from invasive breast cancer. Though one case would not have been recalled using our proposed workflow, the impact of this case on the study is not predictive of the impact of breast lymphomas in a screening situation.

In conclusion, there is a difference in stiffness between malignant and benign lesions in the breast, which this study suggests is possible to detect with MI. Implementing MI in screening can potentially substantially lower recall and biopsy rates. The promising results of this study warrant prospective clinical trials.

## Abbreviations

CC: Craniocaudal
DCIS: Ductal carcinoma in situ
IDC: Invasive ductal carcinoma
ILC: Invasive lobular carcinoma
IQR: Interquartile range
LM: Lateromedial
MI: Mechanical imaging
MLO: Mediolateral oblique
RMPA: Relative mean pressure over lesion area
